# Neural Induction without Mesoderm in Xenopus

**DOI:** 10.1371/journal.pbio.0020149

**Published:** 2004-05-11

## Abstract

xx

Formation of the central nervous system has long been thought to result from an induction process, whereby signals emanating from a portion of the dorsal endomesoderm (the inner middle layer of the developing embryo), known as the Spemann–Mangold organizer, instruct cells of the overlying dorsal ectoderm (outer layer) to become neural instead of epidermal. The Spemann–Mangold organizer was itself defined in Spemann and Mangold's seminal 1924 publication as a portion of the dorsal “vegetal” half (also known as the endodermal, or inner, layer) of a gastrulating Xenopus frog embryo that could induce the differentiation of a whole new axis, including a new central nervous system, when grafted into an abnormal location. (Gastrulation is the process that establishes the basic body plan of the organism as cells arrange themselves into three embryonic germ layers: the endoderm, mesoderm, and ectoderm.) From these and later experiments, the notion emerged that neural induction in Xenopus takes place at gastrulation and requires signals from the mesoderm. (The Spemann–Mangold organizer is itself derived from the endomesoderm.)[Fig pbio-0020149-g001]

**Figure pbio-0020149-g001:**
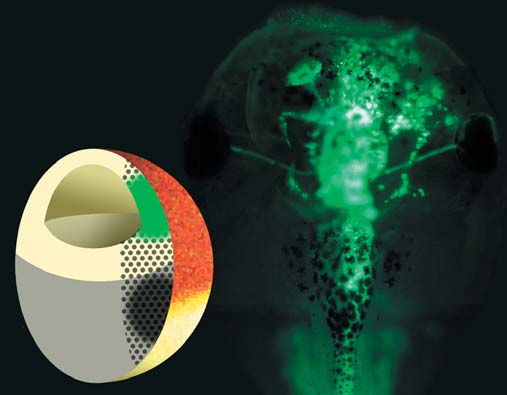
Blastula cells that give rise to the brain

Now Hiroki Kuroda, Oliver Wessely, and Edward De Robertis challenge this model by demonstrating that a group of cells in the dorsal region of the prospective ectoderm is fated to become neuronal as early as the blastula stage (which precedes gastrulation) and that these cells can express their neural character in the absence of any mesodermal influence. The authors call this group of cells the BCNE (blastula Chordin- and Noggin-expressing) center, based on their previous observation that this center expresses the proteins Chordin and Noggin at the blastula stage. Chordin and Noggin are also expressed later in the Spemann–Mangold organizer and are among the key signals that mediate neural induction by the organizer. The presence of the neural inducers in blastula ectodermal precursor cells prompted the authors to test these cells' neural potential. They first demonstrated that BCNE cells normally give rise to the anterior portion of the brain, which confirms these cells' neural fate. Moreover, when cultured in vitro, BCNE cells taken from tissue begin to express neural protein markers, even when extra care is taken to prevent any contact with mesodermal precursors. It therefore appears that BCNE cells are already specified to become neural by the blastula stage, before the Spemann–Mangold organizer forms.

To further demonstrate BCNE cells' independence from mesodermal signals, the authors generate embryos without a mesoderm. Having previously observed that such embryos do develop a central nervous system, Kuroda et al. now demonstrate that this intrinsic neuronal potential depends on Chordin and Noggin expression in BCNE cells. The model that emerges from these experiments suggests that neural induction begins at the blastula stage, with Chordin and Noggin signaling within the BCNE center and may later be consolidated or modulated by signals emanating from the organizer.

What of the endodermal portion of the Spemann–Mangold organizer? It expresses a secreted protein called Cerberus that is involved in development of the head. The authors show that abolishing Cerberus function in the prospective endoderm results in headless embryos. Complete brain removal can also be achieved by partially inhibiting Cerberus function, so long as Chordin is simultaneously inhibited in the dorsal ectoderm. It is therefore likely that while BCNE cells harbor an intrinsic neural potential, neural induction in a living organism occurs via cooperation between the germ layers.

